# Rationale and design of a phase II trial of dacomitinib in advanced non-small cell lung cancer patients with uncommon epidermal growth factor receptor mutations: a prospective and single arm study (DANCE study)

**DOI:** 10.1186/s12885-022-09409-3

**Published:** 2022-03-19

**Authors:** Bo Zhang, Chunlei Shi, Zhiqiang Gao, Hua Zhong, Liwen Xiong, Aiqin Gu, Weimin Wang, Tianqing Chu, Wei Zhang, Huimin Wang, Xueyan Zhang, Runbo Zhong, Baohui Han

**Affiliations:** Department of Pulmonary, Shanghai Chest Hospital, Shanghai Jiao Tong University, 241 West Huaihai Road, Shanghai, People’s Republic of China

**Keywords:** Non-small cell lung cancer, EGFR, Uncommon, Dacomitinib

## Abstract

**Background:**

Dacomitinib is a second-generation, irreversible epidermal growth factor receptor tyrosine kinase inhibitor (EGFR-TKI). ARCHER-1050 showed that this agent can improve progression-free survival and overall survival in advanced non-small cell lung cancer patients with sensitive EGFR mutation compared to gefitinib. However, it is unclear whether dacomitinib is effective in patients with sensitizing uncommon EGFR mutations in exon 18–21. The aim of this study is to investigate the safety and efficacy of dacomitinib in these patients.

**Methods:**

This is a single arm, prospective, open label and phase II trial. Sample size will be calculated by a minimax two-stage design method based on the following parameters: α = 0.075, 1-β = 0.9, P0 = 0.20, P1 = 0.45 and a dropout rate of 10%. A total of 30 eligible patients will be included. Patients will receive continuous oral therapy with dacomitinib (45 mg/day) until disease progression, withdrawal of consent, or unacceptable toxicity, whichever occurs first. The primary endpoint is objective response rate (ORR) per RECIST version 1.1, as assessed by investigators’ review. The second endpoint is disease control rate (DCR), PFS, OS, and safety.

**Discussion:**

We conduct a single arm, phase II study to investigate the safety and efficacy of dacomitinib in advanced NSCLC patients with sensitizing uncommon EGFR mutations. The results of the DANCE study will provide new data regarding efficacy and safety of these patients.

**Trial registration:**

NCT04504071

## Background

About 50% of Asian non-small cell lung cancer (NSCLC) patients have sensitive epidermal growth factor receptor (EGFR) mutations [1, 2]. Several prospective studies have provided robust evidence that tyrosine kinase inhibitors (TKIs) targeting EGFR mutations can not only greatly improve the prognosis of patients compared to traditional chemotherapy, but also greatly improve the quality of life, establishing a new first-line treatment for these patients [3–5]. Currently, almost 200 different EGFR mutation types have been identified. Deletion in exon 19 (19del) and point mutation in exon 21 (21L858R) are the most common, accounting for 85%-90% of the entire EGFR mutation spectrum [1, 6]. In addition to classical mutation (19del or 21L858R), uncommon EGFR mutations were also occasionally identified in clinical practice.

Several retrospective studies investigated the efficacy of first-generation EGFR-TKIs in these patients, but the results have sometimes been controversial due to the small sample size [7–9]. Osimertinib is a third-generation EGFR-TKI. FLAURA study showed that this agent is superior compared with standard first-generation EGFR-TKI in patients with 19del or 21L858R (ORR: 80% vs. 76%, OR = 1.27, 95% CI, 0.85–1.90, *P* = 0.24; PFS: 18.9 m vs. 10.2 m, HR = 0.46, 95% CI, 0.37–0.57, *P* = 0.001) [10], however, for patients with uncommon EGFR mutations, osimertinib is not that impressive. In a single arm, phase II study, 37 advanced NSCLC patients with uncommon EGFR mutation were treated with osimertinib and 61% received osimertinib as first-line treatment. ORR was 50%. Median PFS and DOR were only 8.2 months and 11.2 months, respectively. Median OS was not reached and the 18-months survival rate was 56%. Subgroup analysis showed that patients with S768I poorly response to osimertinib because the ORR was only 38%. [11]. Afatinib is a second-generation EGFR-TKI, a combined post-hoc analysis of LUX-Lung 2/3/6 showed that afatinib was active in advanced NSCLC patients that harbored certain uncommon EGFR mutations. 71.7% patients had ORR and DCR was 84.2%. Median PFS and OS were 10.7 and 19.4 months, respectively. Subgroup analysis suggested that afatinib showed clinical activity in different mutation type. The ORR in patients with G719X, L861Q and S768I were 77.8%, 56.3% and 100%; median PFS were 13.8 months, 8.2 and 14.7 months, respectively; median OS were 26.9 months, 17.1 months and not reached, respectively [12]. Based on these data, afatinib was preferred in patients with uncommon EGFR mutations.

Dacomitinib is an orally taken, irreversible small-molecule inhibitor of EGFR, HER-2 and HER-4. In ARCHER-1050 study, the median progression-free survival (PFS) was 14.7 months in the dacomitinib group, which was statistically superior compared to gefitinib (9.2 months, HR = 0.59, 95% CI 0.47–0.74; *P* < 0.0001). ORR of dacomitinib is 75% [13]. The updated results also suggested that this agent is the first second-generation EGFR-TKI that was demonstrated to improve overall survival (OS) in advanced NSCLC with 19del or 21 L858R (34.1 months vs. 26.8 months, HR = 0.76, 95% CI, 0.58–0.99, *P* = 0.044) [14]. Subgroup analysis suggested that first-line dacomitinib was associated with significant prolongation of PFS and improved OS compared with gefitinib in Asian patients [15]. Adverse event can be well managed with standard medical management and dose modifications [16]. However, it is still unclear whether this agent is effective in patients with uncommon EGFR mutations in exon 18–21. Basic research data suggested that dacomitinib may be active in lung cancer cells with sensitive uncommon EGFR mutation. In lung cancer with exon 18 mutation, the 90% inhibitory concentration of dacomitinib in transfected Ba/F3 cells were lower than the trough concentrations [17]. Case report of a 71-year-old NSCLC woman with 18 G719A showed marked regression when dacomitinib was administered [18]. Based on these results, we proposed our hypothesis that dacomitinib is active for patients with uncommon EGFR mutations.

We conduct this phase II study aiming to investigate the safety and efficacy of dacomitinib in advanced NSCLC patients with uncommon EGFR mutations.

## Methods/Design

### Study design

This is a single arm, prospective, single center, open-label, phase II trial. Eligible patients should begin continuous dacomitinib treatment 3 days after enrollment. Subjects will self-administer dacomitinib 45 mg orally once daily in 21-day cycles until disease progression, withdrawal of consent, death or unacceptable toxicity, whichever occurs first. Treatment option including immunotherapy, was at the investigators’ discretion after disease progression. Dose reductions due to grade 3 or worse treatment-related toxicity or prolonged grade 2 adverse events lasting more than one cycle were permitted. Dacomitinib is available at three dose levels, 45 mg, 30 mg, and 15 mg. Efficacy assessment will be performed every 6 weeks (± 3 days).

**Fig. 1:**
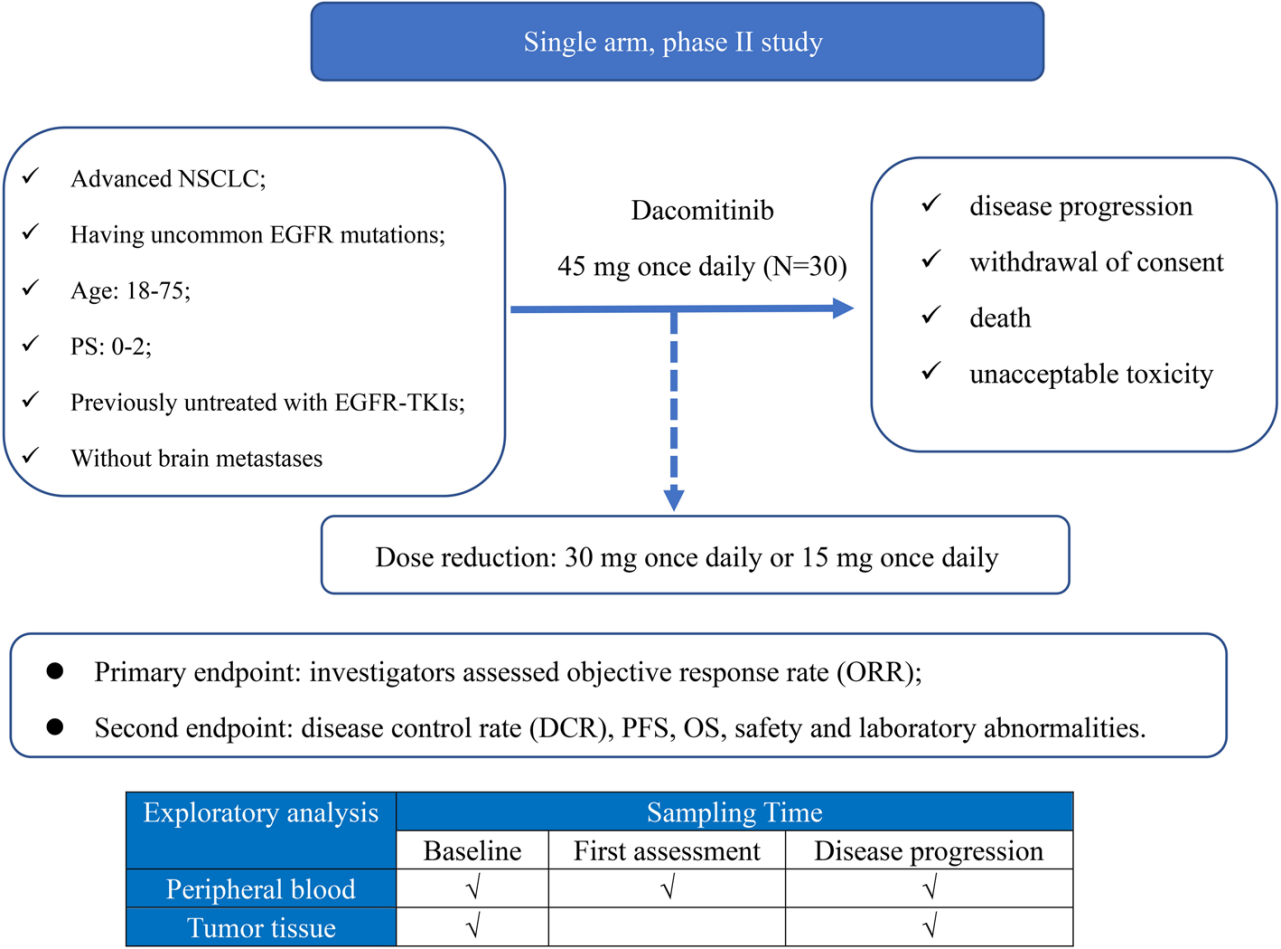
Study schema

The primary endpoint is objective response rate (ORR) per RECIST version 1.1, as determined by investigators’ review. The second endpoint is disease control rate (DCR), PFS, OS, safety and laboratory abnormalities according to the National Cancer Institute Common Terminology Criteria for Adverse Events (CTCAE) v 4.03. Tumor tissue and peripheral blood will be collected at baseline, first assessment (blood only) and disease progression for exploratory analysis (Fig. 1). We have three exploratory endpoints: a) the relationship between ct-DNA clearance at first assessment and response depth; b) drug resistance mechanism; and c) consistency between tissue and baseline peripheral blood. The first patient has been recruited at the end of October, 2021. This study has been registered with clinicaltrial.gov, and the registration number is NCT04504071.

### Inclusion criteria

Only patients who meet all of the following criteria will be enrolled in this study:

(1) According to the 8th edition of the AJCC/UICC TNM staging system for NSCLC, patients with locally advanced (stage III B/III C), metastatic or recurrent (stage IV) NSCLC confirmed by histology or cytology who are unable to undergo surgery and radical concomitant radiochemotherapy and are confirmed to have at least one measurable lesion according to RECIST 1.1.

(2) Patients harboring uncommon EGFR mutations. Uncommon EGFR mutations were defined as mutations in exon 18–21, except for 19del, 21L858R, and well-established drug resistant types (20 insertion, T790M, L747S, L747P, D761Y, T854A). Mutations should be previously reported as sensitive to first- or second-generation TKIs. Detailed mutation types include [19–24].

- Mutation in exon 18: G719X(X = A/C/S/D/E), 18del, E709X(X = G/M/V/H/D/A/K), V689M, S720P/F, P699S, N700D, E709Q, G721A, V740A, L718P;
- Mutation in exon 19: Few exon 19-point mutations with unknown structure and kinase activity have been found in EGFR-TKI responders; however, a new class of sensitizing mutations, exon 19 insertions, were recently found, and those patients were also eligible for this study: I744_K745insKIPVAI, K745_E746insIPVAIK, K745_E746insVPVAIK, K745_E746insTPVAIK.
- Mutation in exon 20: Including S768I, V765A, T783A, V774A, S784P, R776C, R776H, V765M, G779C, G779F, G779S, T783A, T783I, L798F, L798H, K806E, Q812R, L814P.
- Mutation in exon 21: L861Q, R831H, V834I, L838P, L861R.
- Others: Patients with complex mutations but do not have drug-resistant patterns (e.g., 18G719A + 20S768I, 18 E709X + 21L861Q) are also eligible. However, individuals who have common mutations (e.g., 19del + 21L861Q, 18G719X + 21L858R) were not eligible.

(3) Age ≥ 18 years and ≤ 75 years;

(4) Performance status (PS) score: 0 to 2.

(5) Previously untreated with EGFR-TKIs, including first-, second- or third-generation agents. Subjects who were only treated with chemotherapy were eligible, as well as patients who have received adjuvant chemotherapy, but disease recurrence must occur at least 6 months after the last dose of chemotherapy. Palliative radiotherapy must be completed 7 days before the first dose of study drugs;

(6) The main organs function is normal, that is, the following criteria are met:

- Good hematopoietic function, defined as absolute neutrophil count ≥ 1.5 × 10^9^ /L, platelet count ≥ 100 × 10^9^ /L, hemoglobin ≥ 90 g/L [no blood transfusion or no erythropoietin (EPO) dependence within 7 days prior to enrollment]
- Biochemical test results should meet the following criteria: BIL < 1.25 times the upper limit of normal value (ULN); ALT and AST < 2.5 × ULN; in case of liver metastases, ALT and AST < 5 × ULN; Cr ≤ 1.5 × ULN or creatinine clearance (CCr) ≥ 60 ml/min; coagulation function is good, INR and PT ≤ 1.5 times ULN; if the subject is receiving anticoagulant treatment, PT should be within the prescribed range of use of anticoagulant drugs;

(7) Women of child-bearing age should agree to take contraceptive measures (such as intrauterine devices, contraceptives, or condoms) during the study and for 6 months after the study; non-breast-feeding patients whose serum or urinary pregnancy test should be negative; male patients should agree to take contraceptive measures during the study and for 6 months after the study.

(8) Patients are voluntarily enrolled into the study, sign the informed consent form and have good compliance.

### Exclusion criteria

Patients who meet any of the following criteria will be excluded:

(1) Small cell lung cancer (including mixed small cell and non-small cell lung cancer);

(2) Patients who have received EGFR-TKIs as adjuvant or salvaged treatment;

(3) Patients with 19del or 21L858R or well-established drug resistant type;

(4) Patients with many factors affecting oral medication such as dysphagia, gastrointestinal resection, chronic diarrhea, or intestinal obstruction;

(5) Patients who are known to have brain metastases, including asymptomatic metastasis, spinal cord compression, carcinomatous meningitis, or brain or leptomeningeal disease diagnosed by CT or MRI at the time of screening;

(6) Patients with severe and/or uncontrolled diseases, such as:

- Unstable angina pectoris, symptomatic congestive heart failure, myocardial infarction within 6 months before randomization, severe uncontrolled arrhythmias; uncontrolled blood pressure (systolic blood pressure > 140 mmHg, diastolic blood pressure > 90 mmHg);
- Active or uncontrolled serious infection;
- Liver diseases such as cirrhosis, decompensated liver disease, acute or chronic active hepatitis;
- Uncontrolled eye inflammation or eye infection, or any condition that may lead to the above-mentioned ocular diseases;
- Poorly controlled diabetes (fasting blood glucose (FBG) > 10 mmol/L);
- Routine urine test result indicating that urine protein ≥  +  + and 24-h urine protein quantitation is confirmed to be > 1.0 g;
- Active tuberculosis, etc.;
- Uncontrolled hypercalcemia (> 1.5 mmol/L calcium ion or calcium > 12 mg/dL or corrected serum calcium > ULN), or symptomatic hypercalcemia requiring continued diphosphate therapy;
- Long-term unhealed wounds or fractures;

(7) Patients who have a history of psychotropic drug abuse and cannot abstain from it or have mental disorders;

(8) Patients who are known to have severe allergies (≥ grade 3) to active ingredients and any excipients of dacomitinib;

(9) Patients who have other malignant tumors (except radical cervical carcinoma in situ, non-melanoma skin cancer, etc.) at the same time; patients who are evaluated by the investigator to have concomitant diseases that seriously endanger the safety of the patients or affect the patients’ completing the study;

(10) The subjects or their sexual partners cannot or refuse to take effective contraceptive measures during the clinical trial;

(11) Pregnant or breast-feeding women;

(12) Patients in other situations who are evaluated by the investigator to be ineligible to be enrolled.

### Rationale for setting the number of enrolled participants

Sample size will be calculated by a minimax two-stage design method based on the following parameters: α = 0.075, 1-β = 0.9, P0 = 0.20, P1 = 0.45. The optimal two-stage design tests the null hypothesis that *P* ≤ 0.20 versus the alternative that *P* ≥ 0.45. After testing the drug on 12 patients in the first stage, the trial will be terminated if 2 or fewer patients respond. If the trial goes on to the second stage, a total of 27 patients will be studied. If the total number responding is less than or equal to 8, the drug is considered noneffective. Assuming a dropout rate of 10%, 30 patients will be finally enrolled.

### Population to be analyzed

Efficacy will be analyzed based on full analysis set (FAS) and per-protocol set (PPS). Safety will be analyzed based on safety analysis set (SAS).

#### FAS

All the participants enrolled in this study except: (I) patients without informed consent or who retract their informed consent; (II) patients who do not receive any protocol treatment; and (III) patients who don’t have any data after enrollment. Final decisions will be made after discussion with the trial statistician and principal investigator.

#### PPS

All the participants in the FAS except patients with violation of inclusion/exclusion criteria or violation for prohibited concomitant drugs/therapies.

#### SAS

Patients who received at least 1 dose of dacomitinib.

### Statistical methods

Best overall response will be summarized for the FAS population based on the investigator’s assessment. The number and percent of subjects achieving objective responses (CR or PR) will be summarized along with corresponding 2-sided 95% CI using binomial distribution. Median progression-free survival (PFS) and overall survival (OS) will be calculated based on the Kaplan–Meier method. Baseline characteristics, incidence, and severity of adverse events will be summarized.

## Discussion

ARCHER-1050 provided robust evidence that dacomitinib showed superior efficacy compared to first-generation EGFR-TKI in advanced NSCLC with 19del or 21L858R. However, its effectiveness in patients with uncommon EGFR mutations was unclear.

The objective response rate (ORR) of previous publications is heterogeneous, ranging from 15% to 71.7% due to the small sample size and different agent [8, 9, 11, 12, 19, 20]. We have noted the similarly-designed study conducted by Jang Ho Cho and his colleagues, which investigated the safety and efficacy of osimertinib in these patients. In this study, the ORR was 50%, which is numerically superior to 45% [11]; however, median progression-free survival (PFS) should also be compared. In this study, the median PFS was only 8.2 months, which is greatly inferior to that of patients with common EGFR mutation when osimertinib was initiated as a first-line treatment (18.9 months from FLAURA study) [25]. The ORR and median PFS was 71.7% and 10.7 months when treated with afatinib [12]; however, in this combined post-hoc analysis, 36 of 38 patients had widely-known sensitive uncommon EGFR mutations, mainly 18G719X, 20S768I, and 21L861Q. Currently, about 200 different mutations have been reported [6]. The associations among the rest of these mutations (e.g., V689M, S720P/F, P699S, N700D, E709Q, G721A, V740A, L718P) and response to TKIs have not been well established. These patients were eligible for our study. In addition, our previous study suggested that complex EGFR mutations with 19del or 21L858R were sensitive to EGFR-TKIs [26]. These patients were ineligible for our study but were included in the post-hoc analysis (4 patients). That’s why we proposed a relatively conservative hypothesis (P1 = 0.45).

To the best of our knowledge, this is the first study to investigate the safety and efficacy of dacomitinib in these patients. The first patient has been enrolled in December 2020 and is expected to take 26 months.

## Data Availability

Not applicable. Data sharing is not applicable to this article as no datasets were generated or analyzed during the current study.

## Abbreviations

EGFR-TKI: Epidermal growth factor receptor tyrosine kinase inhibitor
PFS: Progression-free survival
OS: Overall survival
ORR: Objective response rate
DCR: Disease control rate
NSCLC: Non-small cell lung cancer
AEs: Adverse events
CTCAE: National Cancer Institute Common Terminology Criteria for Adverse Events
PS: Performance status
FAS: Full analysis set
PPS: Per-protocol set
SAS: Safety analysis set
